# MKP-1 negative regulates *Staphylococcus aureus* induced inflammatory responses in Raw264.7 cells: roles of PKA-MKP-1 pathway and enhanced by rolipram

**DOI:** 10.1038/s41598-017-10187-3

**Published:** 2017-09-28

**Authors:** Yiqing Pan, Chen Xu, Zhixing K. Pan

**Affiliations:** 1 Department of Medical Microbiology and Immunology, University of Toledo College of Medicine, Toledo, OH 43614 USA; 2 Department of Anatomy, Histology and Embryology, Shanghai Jiao Tong University School of Medicine, Shanghai, 200025 China; 3 Shanghai Key Laboratory of Reproductive Medicine, Shanghai, 200025 China

## Abstract

MAP phosphatases (MKP)-1 acts as an important regulator of innate immune response through a mechanism of control and attention both MAPK and NF-κB molecules during bacterial infection. However, the regulatory role of MKP-1 in the interplay between MAPK and NFκB pathway molecules is still not fully understood. In present study, we showed a direct interactions of p38, ERK or IκBα with MKP-1, and demonstrated that MKP-1 was a pivotal feedback control for both MAP kinases and NF-κB pathway in response to *S. aureus*. In addition, we found that rolipram had anti-inflammatory activity and repressed IκBα activation induced by *S*. *aureus via* PKA-MKP-1 pathway. Our report also demonstrated that PKA-cα can directly bind to IκBα upon *S. aureus* stimulation, which influenced the downstream signaling of PKA pathway, including altered the expression of MKP-1. These results presented a novel mechanism of PKA and IκB pathway, which may be targeted for treating *S. aureus* infection.

## Introduction

*Staphylococcus aureus* is a Gram-positive bacterium that is responsible for the vast majority of life-threatening diseases, including serious skin and soft tissue infection, pneumonia, bacteremia, septic arthritis and sepsis^1,2^. *S. aureus* infection is capable of producing systemic cytokine responses. A wide range of inflammatory cytokines and chemokines are produced from blood monocytes and tissue macrophages^3^. It has become apparent that although peptidoglycan (PepG) and lipoteichoic acid (LTA), the major cell wall components of *S. aureus* are recognized by different class of pattern-recognition receptor (NOD or TLR2)^4^, they can trigger the same cascades of signaling events including the activation of the transcription factor NFκB and MAPK pathways, ultimately leading to the production a variety of pro-inflammatory cytokines, including TNFα, IL-1β and IL-6^5^. TNFα is considered one of the key inflammatory mediators and acts as a host defense against bacterial infection. But, overproduction of TNFα also can cause septic shock, multiple organ dysfunction syndrome and inflammatory disorders^6^. Thus, both the induction and termination of pro-inflammatory cytokine production are all crucial for maintaining an appropriate defense during bacterial infection.

Mitogen-activated protein kinase phosphatases (MKPs) belong to a family of dual specificity protein phosphatases, which responsible for dephosphorylation of both phosphothreonine and phosphotyrosine residues^7,8^. MKP-1, the first defined member of MKPs^9^, acts as a crucial negative regulator of the inflammatory response in macrophages during bacterial infection^10^. Liu Y. group demonstrated that MKP-1 could limit the inflammatory reaction to *S. aureus* infection by inactivating MAPK signaling molecules^10^. Meanwhile, an increasing evidence suggested that the induction of MKP-1 could repress NFκB-dependent inflammatory genes expression^11–13^. NFκB may present a possible mechanism of action involved in MKP-1 related negative regulation of inflammatory responses. However, the regulatory role of MKP-1 in the interplay between MAPK and NFκB pathway molecules remains unclear.

The highly selective PDE4 inhibitor rolipram has been used for several years described for its anti-depression property^14^. In addition, anti-inflammatory action of rolipram has been appealed to treatments of autoimmune disorders, such as asthma, chronic obstructive lung disease (COPD)^15^. Several studies have indicated that rolipram could suppress TNFα synthesis and release in response to LPS in the monocytes^16,17^. Meanwhile, MKP-1 was shown to be a negative regulator of the inflammatory responses to different stimuli. It has also reported that MKP-1 was involved in the anti-inflammatory effect of rolipram^17,18^. Therefore, this study was designed to study the contribution of MKP-1 in rolipram associated anti-inflammatory activity by *S. aureus* stimulus.

In the present study, we reported that MKP-1 was a pivotal feedback regulator controlling both MAP kinases and NF-κB pathway. In addition, MKP-1 could directly interact with these molecules at different times, acted as a negative regulator of inflammatory response to *S. aureus*. In addition, we found that rolipram had anti-inflammatory activity through MKP-1 dependent mechanisms. Our results also represented that PKA-cα can directly bind to IκBα in response to *S. aureus*, which influenced the downstream signaling of PKA pathway, including altered the expression of MKP-1. These results brought a novel mechanism between PKA and IκB pathway, which may be targeted for treating *S. aureus* infection.

## Results

### MKPs expressions were enhanced and more robust induction of MKP-1 by *S. aureus* stimulus

**Figure 1:**
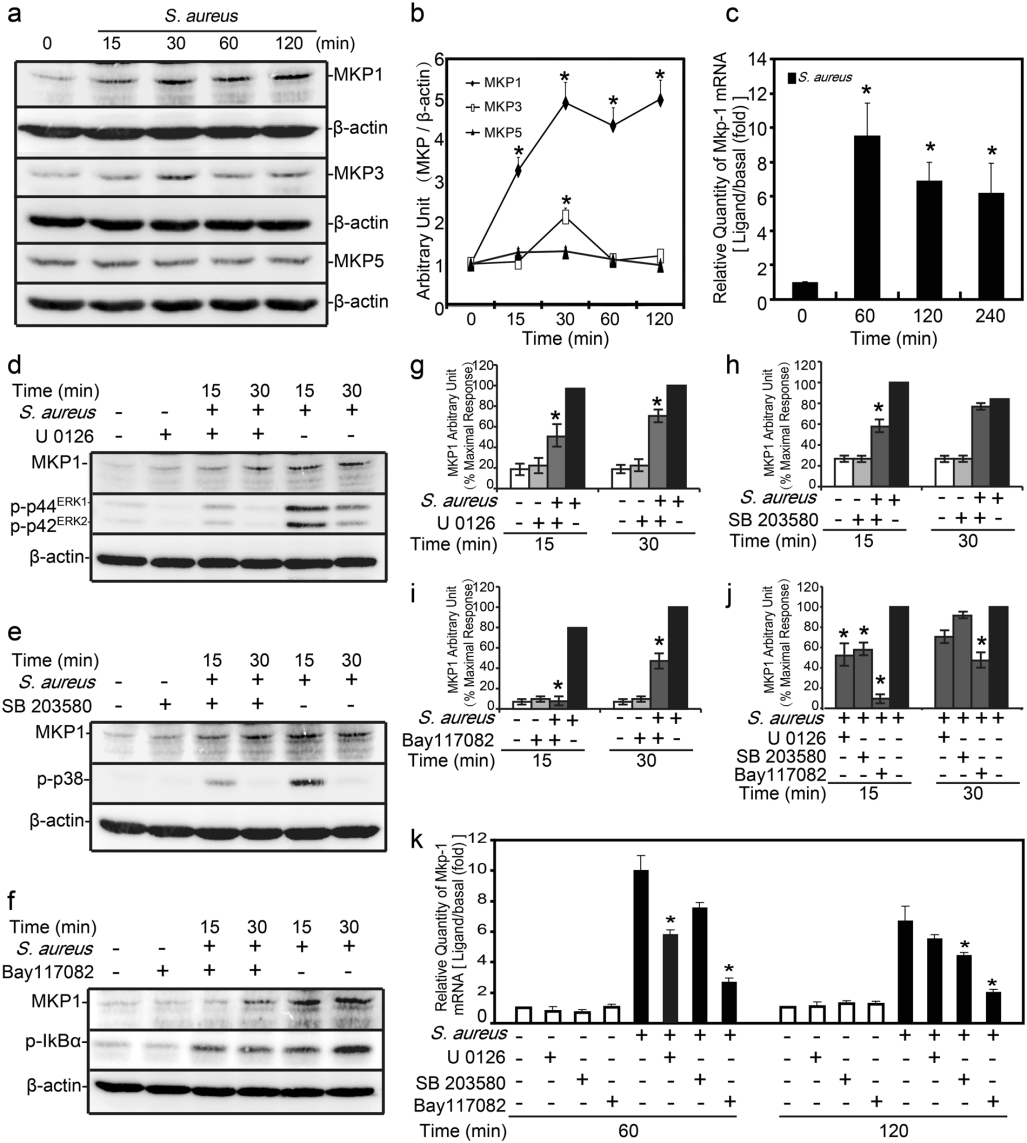
Expressions of MKPs and related signaling molecules induced by *S. aureus* in Raw264.7 cells. (**a**–**c**) Raw264.7 cells stimulated with *S. aureus* (10 M.O.I.) or control. (**a**,**b**) Cells were harvested for protein after 0, 15, 30, 60 and 120 minutes respectively, and cell lysates were subjected to western blot analyses for MKP-1, MKP-3, MKP-5 and β-actin. Immunoblots were scanned, the intensities of bands were quantified by the Image program. Data were normalized to the control, and the ratios were expressed as fold changes with respect to the control samples. (**c**) Cells were harvested for RNA after 60, 120 and 240 minutes, and real-time PCR was carried out using primers specific to *Mkp-1* and *β-actin*. (**d**–**k**) Raw264.7 cells were incubated with U 0126 (10 μM), SB 203580 (10 μM) and Bay 11-7082 (10 μM) as indicated for 30 minutes prior to stimulation with *S. aureus* (10 M.O.I.) or control. (**d**–**j**) Cells were harvested for 15 to 30 minutes, western blot analyses of MKP-1, phospho-ERK1/2, phospho-p38, phospho-IκBα and β-actin were carried out. (**k**) Cells were harvested for 60 to 120 minutes, and real-time PCR was carried out using primers specific to *Mkp-1* and *β-actin*. *p < 0.05.

To determine whether MKP family played a role in regulating of *S. aureus* induced immune response, we first examined the expressions of three distinct subgroups of MKPs: DUSP1/MKP-1, DUSP6/MKP-3 and DUSP10/MKP-5. In response to *S. aureus*, the induction of MKP-1 became evident at 15 minutes, greatly up-regulated from 30 to 120 minutes, and returned to the basal levels at 240 minutes in Raw264.7 cells (Fig. 1a,b). Western-blot results also showed that *S. aureus* increased MKP-3 and MKP-5 expression from 15 to 60 minutes, the peak was at 30 minutes, as shown in Fig. 1a,b. Compared with MKP-3 and MKP-5, MKP-1 was more robust production from 15 to 120 minutes, which indicated that MKP-1 may play a significant role in the innate response by *S. aureus* stimulus. Meanwhile, we assessed the expression of *Mkp-1* at mRNA level in Raw264.7 cells by q-PCR analysis. Similarly, *Mkp-1* induction was increased at transcription level by *S. aureus* stimulus (Fig. 1c).

Increased MKP-1 expression by *S. aureus* stimulus was suppressed by ERK, p38 and IκBα inhibitors. To explore the signaling cascade of MKP-1 expression in *S. aureus* activated Raw264.7 cells, we utilized U 0126 (MEK-ERK1/2 inhibitor), SB 203580 (p38 inhibitor) and Bay 11-7082 (IκBα inhibitor) to assess MKP-1 expression. Since mRNA and protein analyses for MKP-1 revealed that a significant increase was observed from 15 to 120 minutes after *S. aureus* stimulus, this period was used to investigate the effects of these pharmacological inhibitors on the induction of MKP-1 (Fig. 1d–k). Treatments of Raw264.7 cells with U 0126 and SB 203580 (10 μM, pretreatment for 30 minutes) were capable of inhibiting MKP-1 production induced by *S. aureus* at protein level (Fig. 1d,e,g,h,j). Compared with U 0126 and SB 203580, Bay 11-7082 (10 μM, pretreatment for 30 minutes) also had apparent effect on MKP-1 expression upon *S. aureus* stimulation (Fig. 1f–j). Meanwhile, the effects of these pharmacological inhibitors on *Mkp-1* expression at transcription level were also predominant (Fig. 1k). Pretreatments of ERK and p38 inhibitors (10 μM for 30 minutes) suppressed the expression of *Mkp-1* stimulated by *S. aureus* at 60 and 120 minutes respectively (about 0.5 and 0.6 fold). In addition, Bay 11-7082 (10 μM, pretreated for 30 minutes) was capable of inhibiting *Mkp-1* levels from 60 to 120 minutes in response to *S. aureus* stimulation (about 0.3 to 0.4 fold). The effects of these pharmacological inhibitors on MKP-1 expression in Raw264.7 cells revealed that ERK, p38 and IκBα signaling cascades were all involved in MKP-1 synthesis by *S. aureus* stimulus.

### MKP-1 was required for attenuation of *TNFα* production induced by *S. aureus*

**Figure 2:**
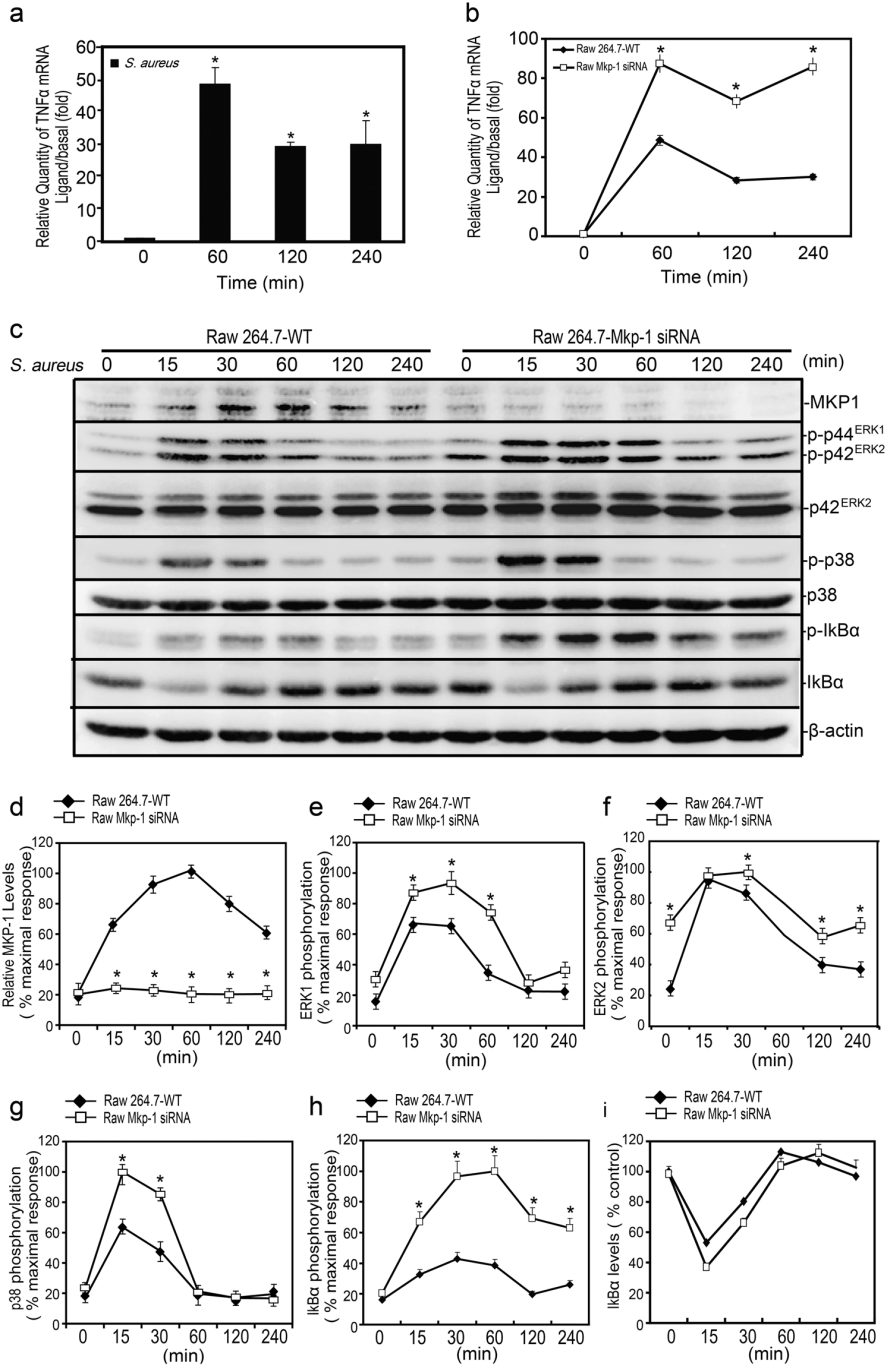
Effect of *Mkp-1* target siRNA on *S. aureus* induced *TNFα* mRNA synthesis and p38, ERK, IκBα phosphorylations in Raw264.7 cells. (**a**) Raw264.7 cells were stimulated with *S. aureus* (10 M.O.I.) or control for 60, 120 and 240 minutes. Cells were harvested, real-time PCR was carried out using primers specific to *TNFα* and *β-actin*. (**b**) Raw264.7 cells stably harboring the *Mkp-1* siRNA and Raw264.7 cells were simulated with *S. aureus* (10 M.O.I.) or control for different time, real-time PCR was performed for *TNFα* and *β-actin*. (**c**) Raw264.7 *Mkp-1* siRNA and Raw264.7 cells were stimulated with *S. aureus* (10 M.O.I.) or control as indicated for 15, 30, 60, 120 and 240 minutes. Cell lysates were subjected to western blot analyses for MKP-1, phospho-ERK1/2 (ERK2), phospho-p38 (p38) and phospho-IκBα (IκBα) and β-actin. (**d**–**i**) Data were normalized to the control and expressed as the percentage of maximum activation of fold change of stimulation. *p < 0.05.

To assess the innate immune response of Raw264.7 cells to *S. aureus* infection, we then determined the effect of *S. aureus* on the pro-inflammatory cytokine-*TNFα* at transcription level. *S. aureus* induced production of *TNFα via* the time-dependent manner (Fig. 2a). Maximal *TNFα* mRNA level was achieved at 60 minutes after stimulation of *S. aureus*, which gradually decreased from 120 to 240 minutes. Our previous study demonstrated that MKP-1 could inhibit *TNFα* production in the macrophages by LPS incubation^17^, we asked whether *Mkp-1* could attenuate the production of *TNFα* by *S. aureus* stimulus. As shown in Fig. 2b, the expression of *TNFα* was increased significantly by siRNA-*Mkp-1* approach. These results indicated that the loss of *Mkp-1* function increased production of *TNFα* induced by *S. aureus* in Raw264.7 cells.

### MKP-1 attenuated *TNFα* production induced by *S*. *aureus via* suppression of ERK, p38 and IκBα activations

Some studies have shown that *S. aureus* or their bacterial components can trigger a cascade of signaling events to activate the NFκB and MAPK pathways, leading to production of a variety of pro-inflammatory cytokines, such as TNFα^4,5,19^. To investigate the activations of ERK, p38 and IκBα on TNFα biosynthesis induced by *S. aureus*, we utilized the pharmacological inhibitors of these signaling molecules to assess TNFα production. As shown in Supplemental Fig. S1, ERK, p38 or IκBα inhibitor (U 0126, SB 203580 or Bay 11-7082, pretreatment for 30 minutes) all decreased the expression of *TNFα* at transcription level at 60 and/or 120 minutes after *S*. *aureus* stimulus (about 0.1 to 0.5 fold). These data demonstrated that ERK, p38 and IκBα molecules were all able to regulate *S. aureus*-mediated *TNFα* production in Raw264.7 cells.

To further explore the role of *S. aureus* induced MKP-1 in the repression of inflammatory molecules, we assessed the expressions of MKP-1 and related signaling molecules in *Mkp-1* knockdown Raw264.7 cells. In Raw264.7 WT cells, *S. aureus* stimulation resulted in an increase in MKP-1 protein level, which temporally coincided with the inactivations of ERK1/2 and p38. No MKP-1 protein was detected in *Mkp-1* siRNA cells when stimulated by *S. aureus* (Fig. 2c,d). Gene silencing of *Mkp-1* by RNAi in Raw264.7 cells exhibited a substantial increase and prolonged (from 15 to 60 minutes) ERK1/2 activation compared with WT cells (Fig. 2c,e,f). In addition to the total ERK2, our western-blot analyses revealed an immunoreactive band of slightly higher molecular weight. The manufacturer’s data sheets of ERK2 (Santa Cruz Biotechnology, USA) suggested this band should be ERK1, we would not comment further on this band. Consistent with the sustained ERK1/2 activation in *Mkp-1* siRNA cells, *S. aureus* stimulation resulted in an increased p38 activation (at 15 and 30 minutes) in *Mkp-1* siRNA cells. However, our results showed that there was no difference in duration of p38 activation between 60 and 240 minutes by *S. aureus* stimulus (Fig. 2c,g). Interestingly, MKP-1 was also involved in attenuation of IκBα activation induced by *S. aureus*. Maximal expression of phospho-IκBα was detected at 30 minutes after *S. aureus* stimulation and gradually returned to the basal level till 120 minutes. *Mkp-1* targeting siRNA produced an increased, earlier and prolonged (from 15 to 240 minutes) IκBα activation, but the total IκBα protein levels were not changed when treated with *S. aureus* (Fig. 2c,h,i). Meanwhile, our previous data demonstrated that *Mkp-1* targeting siRNA had a significant increase in the production of *TNFα*. In addition, ERK, p38 and IκBα molecules were all involved in regulating *S. aureus*-mediated *TNFα* production in Raw264.7 cells. Considered together, it was possible that *S. aureus* induced MKP-1 could inhibit IκBα, ERK and p38 mediated cytokines production by suppression of their activations.

### PKA pathway was involved in MKP-1 production induced by *S. aureus*

**Figure 3:**
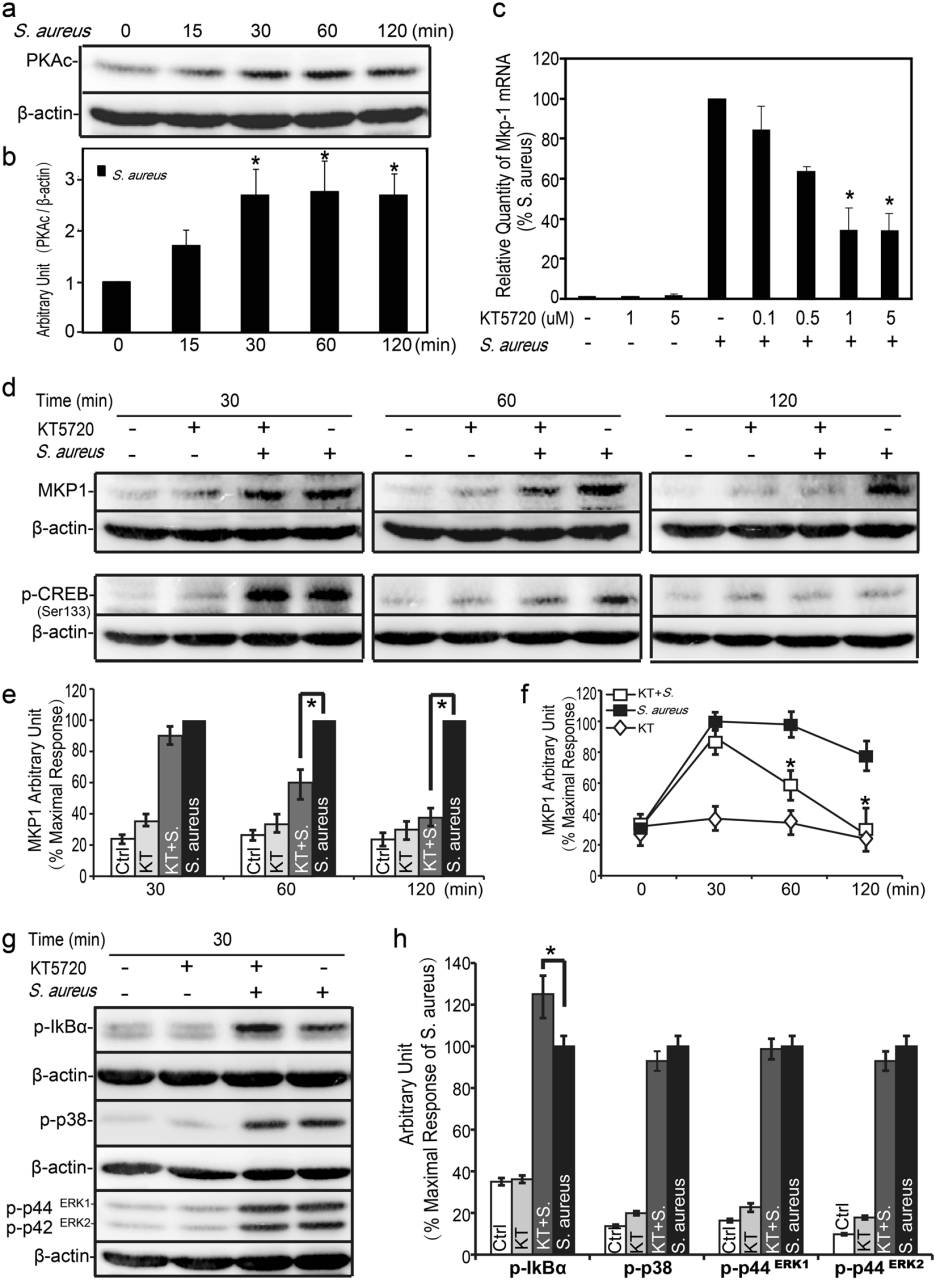
Involvement of PKA-c activation in *S. aureus* induced MKP-1 production. (**a**,**b**) Raw264.7 cells were either not stimulated or stimulated with *S. aureus* (10 M.O.I.). Cells were harvested for protein after 0, 15, 30, 60 and 120 minutes, and cell lysates were subjected to western blot analyses for PKA-cα and β-actin. (**c**) Raw264.7 cells were incubated with KT-5720 (0.1~5 μM) as indicated for 30 minutes prior to stimulation with *S. aureus* (10 M.O.I.) for 60 minutes. Cells were harvested for RNA, and real-time PCR was carried out using primers specific to *Mkp-1* and *β-actin*. (**d**–**h**) Raw264.7 cells were incubated with KT-5720 (1 μM) as indicated for 30 minutes prior to stimulation with *S. aureus* (10 M.O.I.). Cell lysates were subjected to western blot analyses for MKP-1, phospho-CREB, phospho-IκBα, phospho-p38 and phospho-ERK1/2 for indicated time. Immunoblots were scanned, the intensities of bands were quantified by the Image program. Data were normalized to the control, and the ratio was expressed as fold change with respect to the control sample or as the percentage of maximum activation of stimulation. *p < 0.05.

Several lines of evidence indicated that PKA pathway was involved in the innate immune response and the cytokine release when stimulated with the PepG and LTA, the pure cell wall constituents of *S. aureus* ^20,21^. Meanwhile, some studies have shown that PKA catalytic subunit (PKA-c) was involved in the inflammation during bacterial infection in macrophages^22–25^. Then we explored the effect of *S. aureus* on PKA-cα (one catalytic isoform of PKA-c) expression in Raw264.7 cells. As shown in Fig. 3a,b, *S. aureus* treatment induced a substantial high level expression of PKA-cα, which greatly up-regulated at 30 minutes, reached a maximum value at 60 minutes and decreased a little at 120 minutes.

Some studies have reported that MKP-1 was involved in the PKA signaling when treated with rolipram^17,18^, we hypothesized that *S*. *aureus* induced MKP-1 could be regulated by PKA pathway. We took advantage of a specific inhibitor for PKA, KT-5720 and assessed MKP-1 expression by *S. aureus* stimulus. Since the peak of *S. aureus* induced *Mkp-1* mRNA expression occurred at 60 minutes, this period was used to study the effect of KT-5720 on the induction of *Mkp-1*. As shown in Fig. 3c, when pre-treated Raw264.7 cells with KT-5720 (0.1~5 μM, 30 minutes) could inhibit *S. aureus* induced *Mkp-1* transcription *via* the dose-dependent manner. KT-5720 (1 μM and 5 μM) suppressed *S. aureus* induced *Mkp-1* production at 60 minutes (about 0.3 fold).

CREB is known to be one of the major downstream molecule in PKA pathway. PKA is able to activate CREB by phosphorylation at Ser133. CREB (Ser133) has been used to monitor the activation of PKA in numerous studies^26^. *S. aureus* stimulation induced a transient activation of CREB in Raw264.7 cells (up-regulated at 30 minutes, decreased at 60 minutes, returned to the basal level at 120 minutes, as shown in Fig. 3d). KT-5720 (1 μM, 30 minutes) attenuated *S. aureus* induced CREB (Ser133) phosphorylation at 60 minutes. In addition, *S. aureus* induced MKP-1 expression was also suppressed by KT-5720 (Fig. 3d–f) at 60 minutes (0.6 fold) and 120 minutes (0.3 fold). Taken together, it suggested that *S. aureus* induced MKP-1 production could be regulated by PKA pathway in Raw264.7 cells.

### Rolipram repressed *TNFα* production induced by *S. aureus* as well as the activation of related signaling molecules

**Figure 4:**
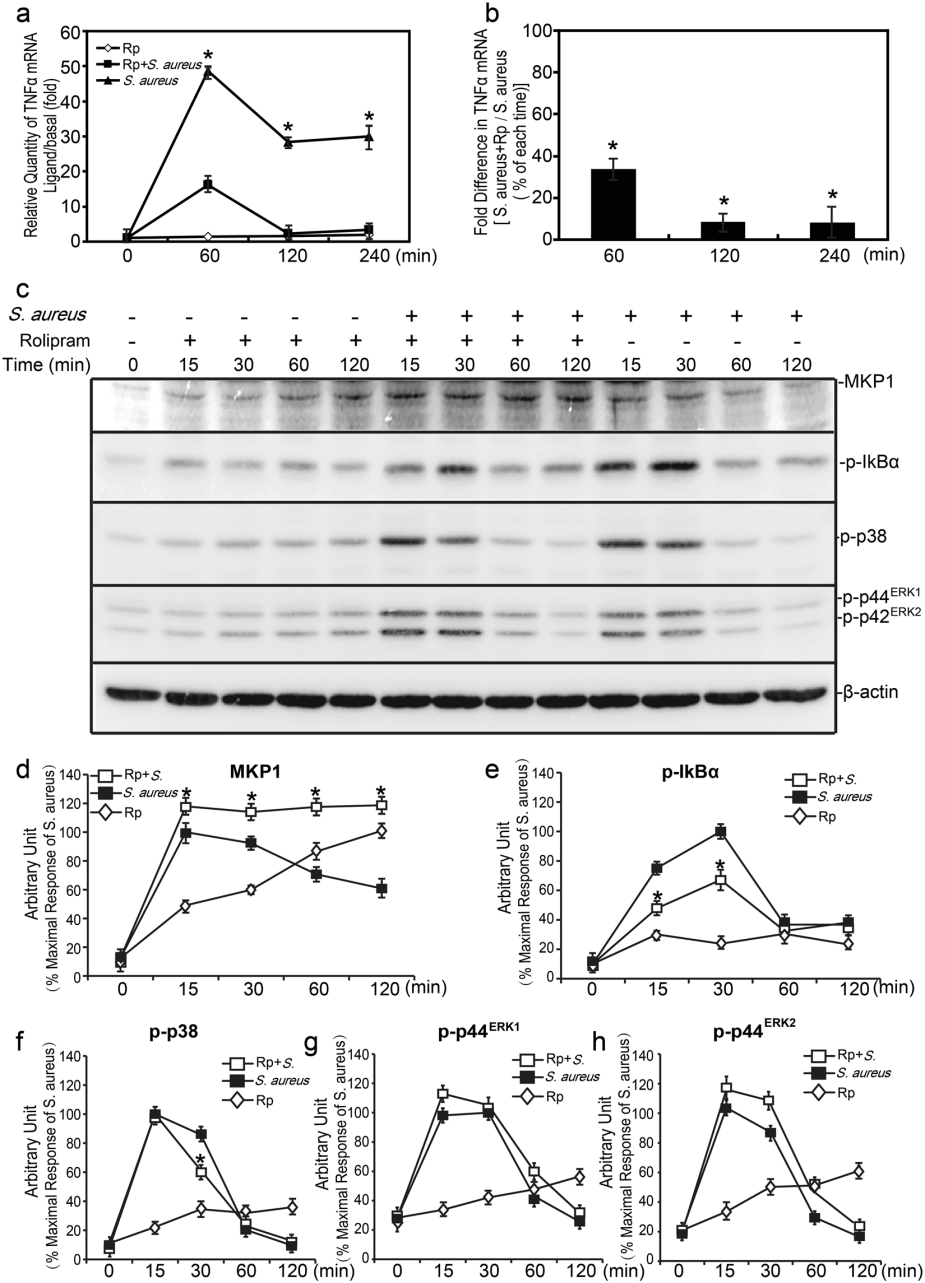
Effect of rolipram on *TNFα* production and related signaling molecules activation induced by *S. aureus* in Raw264.7 cells. Raw264.7 cells were incubated with rolipram (10 μM) for 30 minutes prior to stimulation with *S. aureus* (10 M.O.I.) or control. (**a**,**b**) Cells were harvested for 60, 120 and 240 minutes. Relative quantity and percentage of *TNF* mRNA expression was measured by real-time PCR analysis. (**c**) Cells were harvested for 15, 30, 60 and 120 minutes, MKP-1, phospho-IκBα, phospho-p38 and phospho-ERK1/2 were measured by western-blot analyses. (**d**–**h**) The chemiluminescent signal was normalized to the control and expressed as the percentage of maximum activation of stimulation. *p < 0.05.

Among the large phosphodiesterases (PDEs) family, PDE4 is mainly expressed in neutrophils, macrophages and T cells. An increasing number of data now indicated that the PDE4 inhibitor had the negative modulatory effects on the inflammatory responses, including inflammatory cells activation and cytokines release^15–18^. We thus utilized PDE4-specific inhibitor, rolipram to pre-treat Raw264.7 cells, and then assessed the production of TNFα induced by *S. aureus* stimulus. Rolipram (10 μM, pretreated for 30 minutes) could inhibit *S. aureus* induced *TNFα* expression at the mRNA level in Raw264.7 cells (Fig. 4a,b). Then we explored the effects of rolipram on TNFα expression related signaling molecules. As shown in Fig. 4c,e,f, phosphorylations of p38 and IκBα were increased in response to *S. aureus* stimulus and they were reduced obviously by rolipram. Compared with p38 and IκBα, rolipram had little effect on the activation of ERK1/2 (Fig. 4c,g,h). The inhibitory effects of rolipram on *TNFα* production and related signaling molecule activations indicated that rolipram could inhibit inflammatory gene expression and inflammation induced by *S. aureus*.

### Rolipram enhanced MKP-1 expression in resting and activated Raw264.7 cells

**Figure 5:**
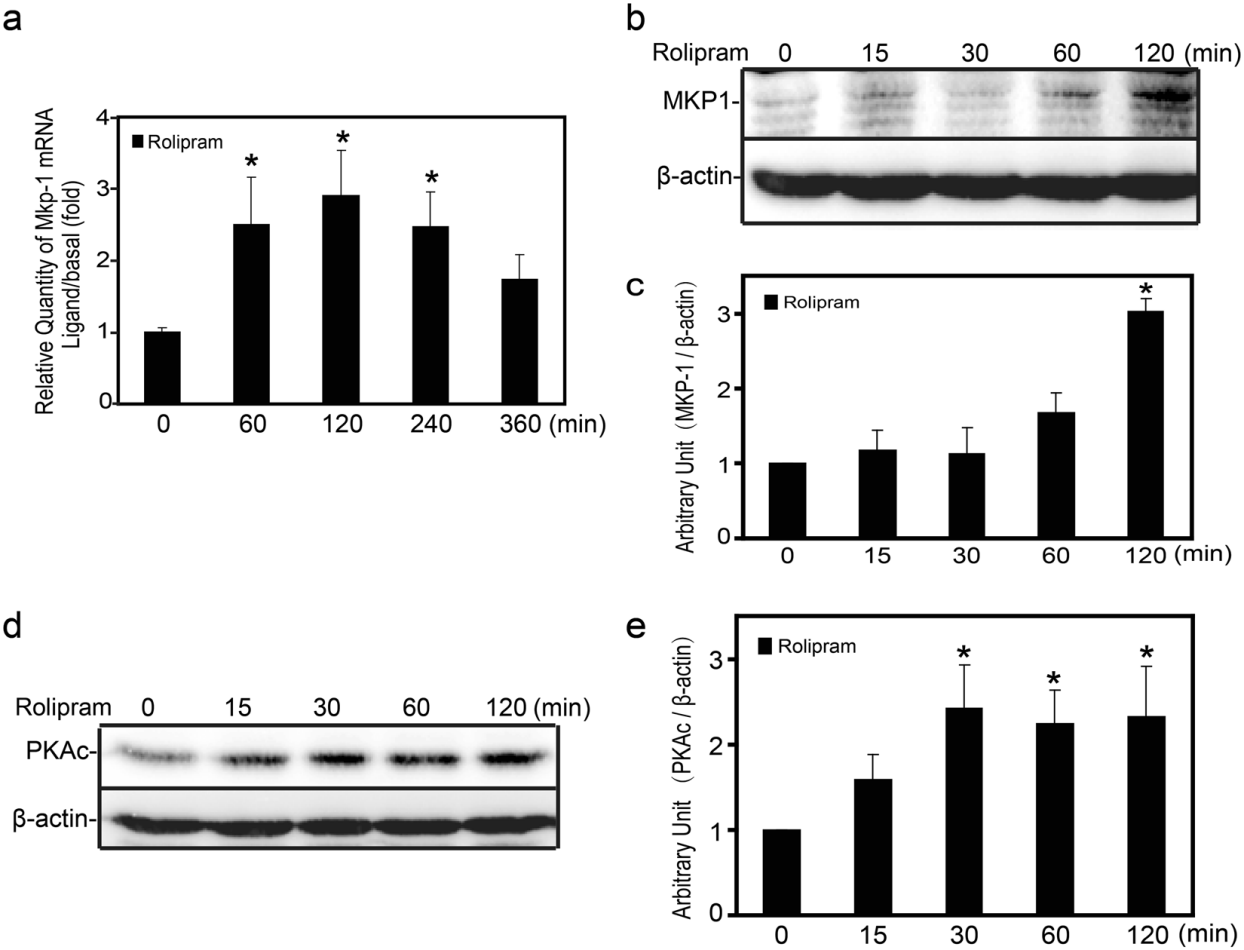
Effects of rolipram on MKP-1 and PKA-cα expression in Raw264.7 cells. (**a**) Raw264.7 cells were treated with rolipram (10 μM) for 60, 120 and 240 minutes. Cells were harvested for RNA, and real-time PCR was carried out using primers specific to *Mkp-1* and *β-actin*. Cells were treated with rolipram (10 μM). for 0, 15, 30, 60 and 120 minutes, and cell lysates were subjected to western blot analyses for MKP-1 (**b**,**c**), PKA-cα (**d**,**e**) and β-actin. Immunoblots were scanned, the intensities of bands were quantified by the image program. Data were normalized to the control, and the ratio was expressed as fold change with respect to the control sample. *p<0.05.

To evaluate the contribution of MKP-1 in rolipram-mediated anti-inflammatory effects, we assessed MKP-1 expression in Raw264.7 cells after treated with rolipram. As shown in Fig. 4c,d, MKP-1 expression was increased by *S. aureus* stimulus and it was further enhanced in the presence of rolipram in Raw264.7 cells. In addition, induction of MKP-1 was increased obviously at both transcription and translation levels by administration of rolipram alone in the Raw264.7 cells (Figs 4c,d and 5a–c). Our previous data demonstrated that MKP-1 could be a major feedback regulator of innate immune response and suppress ERK, p38 and IκBα mediated *TNFα* production. Taken together, these data indicated that rolipram could enhance MKP-1 expression and MKP-1 may be involved in the anti-inflammatory effects of rolipram by *S. aureus* stimulus.

### Rolipram up-regulated MKP-1 expression *via* cAMP-PKA dependent signaling pathway

It has been demonstrated that PDE4 is a cAMP-specific PDE which is expressed in the inflammatory cells, inhibition of PDE4 by rolipram can lead to an elevation of endogenous cAMP levels and down-regulation of the inflammatory response^18^. To investigate the role of cAMP in rolipram induced anti-inflammatory effects, we checked endogenous cAMP levels in our experiments by rolipram incubation. We also challenged the Raw264.7 cells with the cAMP-elevating agents such as forskolin (activator of adenylyl cyclase) and 8-Bromo-cAMP (activator of protein kinase A) as the positive control. Compared with forskolin and 8-Bromo-cAMP, rolipram displayed modest but predominant effects on cAMP levels in Raw264.7 cells (Supplemental Fig. S2).

Some studies have shown that cAMP-dependent suppression of TNFα production is mediated *via* PKA-cα in macrophages by LPS stimulation^22–25^. To further investigate the role of PKA-cα in rolipram induced anti-inflammatory effect, we assessed the expression of PKA-cα in Raw264.7 cells after treating with rolipram. As shown in Fig. 5d,e, treatment with rolipram substantially increased PKA-cα expression in the time-dependent manner, which suggested that the involvement of cAMP-PKA signaling in rolipram mediated anti-inflammatory effects.

**Figure 6:**
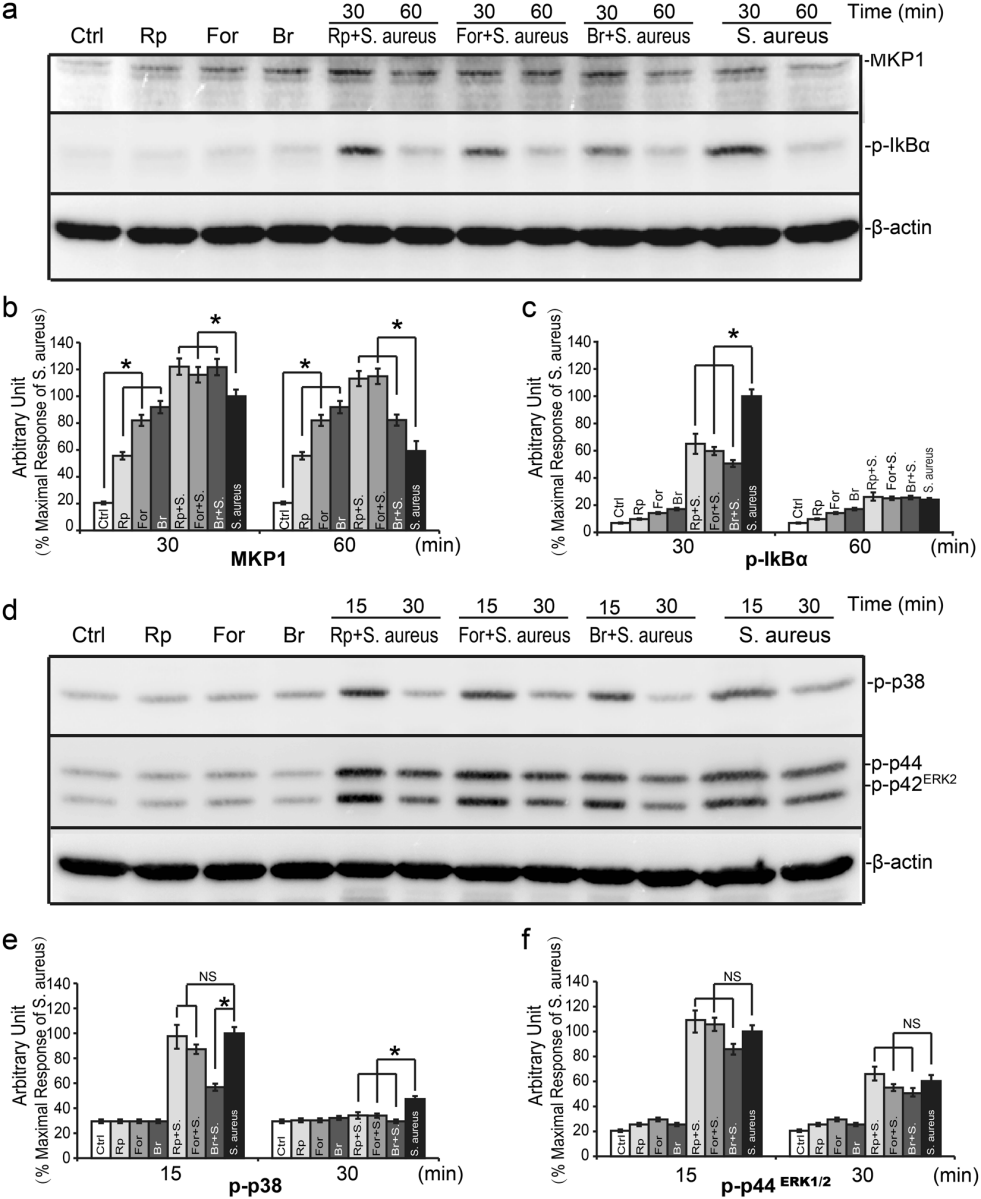
Effects of rolipram, foskolin and 8-Bromo-cAMP on MKP-1 expression and p38, ERK, IκBα phosphorylations induced by *S. aureus* in Raw264.7 cells. Raw264.7 cells were pre-incubated with rolipram (10 μM), foskolin (10 μM) and 8-Bromo-cAMP (250 μM) for 30 minutes prior to stimulation with *S. aureus* (10 M.O.I.) or control. (**a**,**d**) MKP-1/phospho-IκBα (30, 60 minutes) and phospho-p38/phospho-ERK1/2 (15, 30 minutes) were measured by western-blot analyses. The chemiluminescent signal was normalized to the control and expressed as the percentage of maximum activation of stimulation (**b**,**c**,**e**,**f**). *p < 0.05.

Since there is convince evidence that MKP-1 expression could be regulated by cAMP-PKA pathway^17,18,27^, we then sought to determine the effect of cAMP-PKA pathway on MKP-1 expression induced by *S. aureus* in Raw264.7 cells. We utilized the cAMP-elevating agents to assess the expression of MKP-1 in Raw264.7 cells by *S. aureus* stimulus. As shown in Fig. 6a,b, the expression of MKP-1 was significant induced by forskolin and 8-Bromo-cAMP alone or in combination with *S. aureus* in Raw264.7 cells. Similar to the cAMP-elevating agents, *S. aureus-*stimulated MKP-1 expression was increased obviously by rolipram, and it was further enhanced in the presence of rolipram in Raw264.7 cells. Taken together, these data indicated that PDE4 inhibitor rolipram could enhance the expression of MKP-1, and at least partly, by cAMP-PKA dependent pathway.

### Rolipram repressed IκBα activation induced by *S*. *aureus via* cAMP-PKA pathway

Our previous data showed that the anti-inflammatory effect of rolipram on *S. aureus* induced inflammation was mediated by MKP-1 via cAMP-PKA pathway. We also found that MKP-1 was required for attenuation of inflammation induced by *S*. *aureus via* suppression of IκBα, p38 and ERK activations. We hypothesized that IκBα, p38 and ERK molecules may involve in attenuation of inflammation caused by rolipram *via* cAMP-PKA pathway. In order to verify the hypothesis, we utilized the rolipram and cAMP-elevating agents to assess the activated forms of IκBα, ERK and p38 in Raw264.7 cells by *S. aureus* stimulus. As shown in Fig. 6a,c, treatments of Raw264.7 cells with rolipram, forskolin or 8-Bromo-cAMP were all capable of inhibiting IκBα activation induced by *S. aureus* at 30 and 60 minutes. These agents also had obvious effects on p38 activation induced by *S. aureus*, resulting in ~40% reduction in p38 phosphorylation at 30 minutes (Fig. 6d,e). Meanwhile, prior to incubation with rolipram and other agents revealed modest effect on ERK activation (Fig. 6d,f). Therefore, these data demonstrated that IκBα and p38 molecules could be regulated by increasing intracellular cAMP concentration in Raw264.7 cells.

Because PKA represents the major downstream signaling effector of cAMP, we continued by investigating the effect of PKA inhibitor (KT 5720) on *S. aureus* induced activations of IκBα, p38 and ERK in Raw264.7 cells. As shown in Fig. 3g,h, treatment of Raw264.7 cells with KT-5720 (1 μM, pretreated 30 minutes) markedly enhanced the activation of IκBα by *S. aureus* stimulus. Meanwhile, in response to *S. aureus*, administration of KT-5720 had little effects on p38 and ERK phosphorylations. Furthermore, our previous results showed that KT-5720 could inhibit *S. aureus* induced MKP-1 production. Taken together, these data indicated that the interaction between MKP-1 and IκBα by *S. aureus* stimulus could be regulated *via* cAMP-PKA pathway.

### Interactions of ERK, p38 and IκBα with MKP-1

**Figure 7:**
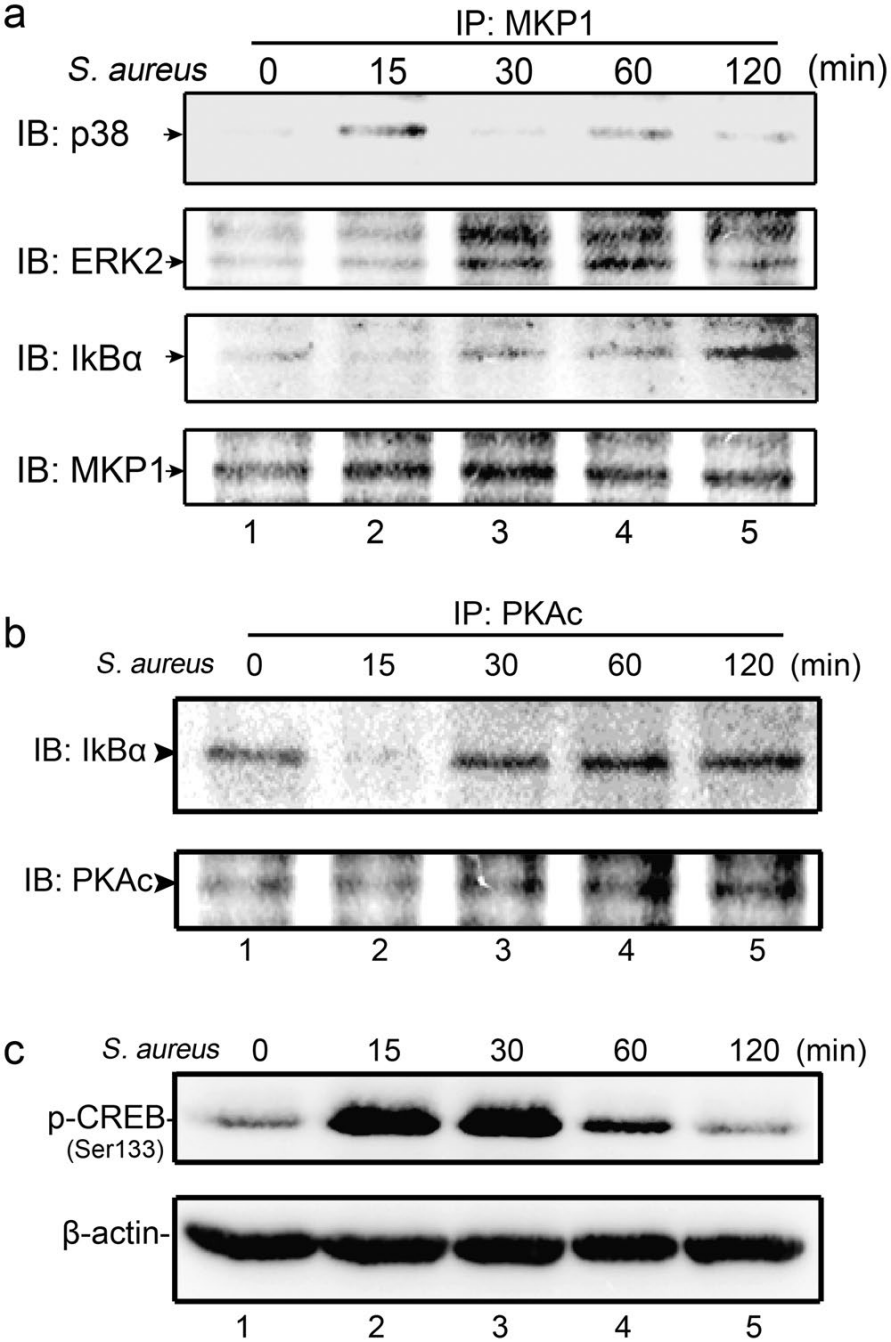
Interactions of p38, ERK, IKBα with MKP-1 and PKA-cα, directly associated with IKBα. Raw264.7 cells were either not stimulated or stimulated with *S. aureus* (10 M.O.I.). Cells were harvested for protein after 0, 15, 30, 60 and 120 minutes respectively. (**a**) MKP-1 was immunoprecipitated (IP) from cell lysates and analyzed by western blotting for presence of p38, ERK, IKBα and MKP-1. (**b**) PKA-cα was IP from cell lysates and analyzed by western blotting for presence of IKBα and PKA-cα. (**c**) Cell lysates were subjected to western blot analyses for phospho-CREB and β-actin.

It has been shown that MKP-1 expression can be up-regulated through the interaction with p38 and ERK molecules using biochemical methods^28,29^. Our previous studies also suggested that knockdown of *Mkp-1* in Raw264.7 cells resulted in a substantial increase of p38 and ERK phosphorylations by *S. aureus* stimulus. We explored the possibility of a direct interaction between MKP-1 and p38 in Raw264.7 cells by *S. aureus* stimulus. After stimulating the Raw264.7 cells by *S. aureus* at different times, the immunoprecipitation was performed using antibody against MKP-1 (Fig. 7a). The precipitates were fractionated by SDS-PAGE and immunoblotted with antibody against p38. In the resting cells, p38 was not linked to MKP-1 (Fig. 7a, lane 1). After stimulated with *S. aureus*, p38 was efficiently precipitated with MKP-1 at 15 minutes, then this interaction was decreased (Fig. 7a, lane 2–5). We also assessed the ability of MKP-1 to bind ERK directly. As shown in Fig. 7a, ERK was sufficiently detected in precipitates of MKP-1 at 30 and 60 minutes (lane 3–4). It was not the case in the resting cells, indicating that there was a specific affinity between MKP-1 and ERK after stimulated with *S. aureus*.

Our previous data demonstrated that IκBα activation was markedly enhanced in siRNA-*Mkp-1* Raw264.7 cells by *S. aureus* stimulus. We hypothesized that MKP-1 may exert its effect through direct interaction with IκBα in Raw264.7 cells. We thus tested our hypothesis by assessing the interaction between MKP-1 and IκBα. Immunoprecipitation in our study suggested that IκBα could precipitate with MKP-1 at 120 minutes after *S. aureus* stimulation (Fig. 7a, lane 5). Interestingly, although direct interactions of p38, ERK or IκBα with MKP-1 were detected in Raw264.7 cells stimulated by *S. aureus*, they exhibited different peak time when linking to MKP-1. Taken together, these results indicated that MKP-1 could modulate MAPK pathway and NFκB cascade by direct interactions with these molecules (p38, ERK and IκBα) at different times. Thus, MKP-1 could be acted as a negative regulator for *S. aureus* induced inflammatory response.

### PKAc, directly associates with IKBα

The data above showed that MKP-1 could control IκBα by direct interaction and thus it might be modulated by cAMP-PKA pathway. In addition, some other groups demonstrated that PKA-cα, but not PKA-cβ, PKA-cγ nor any of R subunit could bind specifically to the cytosolic IκBα and regulated the activity of NFκB^30^. This interaction was further confirmed by another study on platelet activation by thrombin and collagen stimulation^31^. We then explored a direct interaction between PKA-cα and IκBα by *S. aureus* stimulus. As the procedures described above, experiments using the antibody against PKA-cα, the immunoprecipitates were fractioned and immunoblotted with antibody against IκBα. IκBα was detected at the resting cells (Fig. 7b, lane 1). This result was consisted with the studies in other cells^30,31^. After stimulated by *S. aureus* for 15 minutes, the binding between PKA-cα and IκBα was remarkably decreased, almost could not be detected (Fig. 7b, lane 2), which suggested stimulation of Raw264.7 cells with *S. aureus* activated NFκB, at least in part through IκBα-dependent pathway, phosphorylated IκBα, disrupted the PKAcα-IκBα complex, and released the free, active PKA-cα. At the same time, CREB, the major downstream molecule in PKA pathway, was greatly activated by phosphorylation at Ser133 (Fig. 7c, lane 2). Additionally, IκBα was re-detected at 30 minutes by *S. aureus*, more efficiently precipitated at 60 minutes, and decreased a little at 120 minutes. These data indicated that the PKAcα-IκBα complex re-constituted by *S. aureus* stimulation from 30 to 120 minutes. Consistent with these results, PKA-cα expression was greatly up-reregulated in this period (Figs 6a and 7b, lane 3–5). To further explored whether this interaction would modulate the activity of PKA, we assessed the expression of CREB (Ser133). In contrast, the phosphorylation at Ser133 of CREB was sharply decreased at 60 minutes, returned to the basal level at 120 minutes by *S. aureus* in Raw264.7 cells (Fig. 7c, lane 4–5). These results demonstrated that IκBα associated with PKA-cα suppressed the catalytic activity of PKA, and influenced the downstream signaling of PKA pathway. Our report may present a novel mechanism on PKA and IκB pathway in an auto-regulatory feedback manner by *S. aureus* stimulus in Raw264.7 cells.

## Discussion

The present study demonstrated that MKP-1 acted as a negative regulator of inflammatory response to *S*. *aureus via* controlling both MAP kinases and NF-κB molecules. In addition, we found that the anti-inflammatory effect of rolipram on *S. aureus* induced inflammation was mediated by MKP-1 *via* cAMP-PKA pathway. Furthermore, we uncovered the relationship between PKA-cα and IκBα in response to *S. aureus*, and our study provided a novel mechanism between MKP-1 and anti-inflammatory effect of rolipram by *S. aureus* stimulus in Raw264.7 cells.

A major function of the innate immune cells during microbial infection is to detect the pathogen-associated molecular patterns through their specialized receptors and trigger an evolutionarily signaling cascades, which in turn induce the production of pro-inflammatory cytokines and chemokines^4,5,19^. On the other hand, a variety of negative regulator could modulate the strength/duration of the signals and control the production of inflammatory cytokines^6^. MKPs has been shown to act as a crucial negative regulator of the inflammatory response in macrophages during different stimuli^10^. In our study, MKP-1 was robust induced by *S. aureus* stimulus. In addition, the production of *TNFα* was greatly increased when using the *Mkp-1* targeting siRNA approach, which implicated that MKP-1 could play an important role in response to *S. aureus* stimulation. Previous studies have demonstrated that MKP-1 could be rapidly up-regulated and appeared to exhibit a broad substrate specificity in some infection status^9^, we then assessed the effects of different pharmacological inhibitors on MKP-1 expression induced by *S. aureus* stimulus in Raw264.7 cells. In present study, *S. aureus* induced a transient activation of MAPK and NFκB molecules. Treatments of Raw264.7 cells with the ERK, p38 and IκBα inhibitors sharply attenuated *S. aureus* induced MKP-1 production at both mRNA and protein levels. Meanwhile, knockdown of *Mkp-1* in Raw264.7 cells exhibited the substantial increase and/or prolonged the p38, ERK and IκBα activations compared with WT cells. Furthermore, the immunoprecipitation experiments showed that a direct interactions of p38, ERK or IκBα with MKP-1 at different times in Raw264.7 cells by *S. aureus* stimulus. Our data suggested that MKP-1 would be a pivotal feedback control both MAP kinases and NF-κB pathway in response to *S. aureus*. This finding was consistent with previous studies indicating that MAPK and NFκB molecules were all associated with MKP-1 dependent negative regulation^12,32^. Taken together, it suggested that various signaling components were involved in the inflammatory response by *S. aureus* stimulus, they cooperated each other and assessed a distinct spatio-temporal regulation. MKP-1 acted as an important negative regulator for the signaling cascades, strictly controlled the duration and intensity of the inflammatory response to *S. aureus*.

It has been reported that *Mkp-1* is a target gene regulated by CREB regulon^33^. MKP-1 could be regulated by PKA-CREB pathway^18,26,27^. We then assessed the induction of MKP-1 and PKA-CREB pathway molecules by *S. aureus* infection. In response to *S. aureus*, MKP-1 and PKA-cα expression was greatly up-regulated. In addition, *S. aureus* stimulation induced a transient activation of CREB (Ser133). Furthermore, PKA inhibitor, KT-5720 blocked the activation of CREB (Ser133) and production of MKP-1 at the same time by *S. aureus* stimulus in Raw264.7 cells. All these results suggested that increase of MKP-1 expression to *S. aureus*, at least in part, by a mechanism dependent on PKA-CREB pathway.

Rolipram, a classic PDE4-specific inhibitor which often used to treat for asthma, chronic obstructive lung disease^15^. A number of mechanisms have been proposed for the anti-inflammatory actions of rolipram, including repression of inflammatory cytokines releases^16^. Meanwhile, some studies suggested MKP-1 was involved in the anti-inflammatory effects by rolipram^17,18^. However, the molecular mechanisms underlying the role of MKP-1 in anti-inflammatory effects by rolipram remain unclear. Since rolipram treated always results in an elevation of intracellular cAMP levels in the cells^14–16^. Additionally, it has been shown that the primary mediator of the cellular response to cAMP is the PKA^22,24^. We first testified that rolipram could substantially inhibit the production of TNFα by *S. aureus* stimulus. In addition, rolipram could increased the intracellular cAMP levels in Raw264.7 cells. Meanwhile, PKA-cα was greatly induced by rolipram treated. These data indicated that rolipram could suppress *S. aureus* induced immune response by cAMP-PKA pathway. To further evaluate the contribution of MKP-1 in rolipram mediated anti-inflammatory effect, we assessed the MKP-1 expression after treating Raw264.7 cells with *S. aureus* or combined with rolipram. Our finding suggested that MKP-1 was involved in rolipram associated anti-inflammatory effect *via* a cAMP-PKA dependent pathway by *S. aureus* stimulus. This notion was supported by the data as follows. First, induction of MKP-1 was increased obviously at both transcription and translation levels by administration of rolipram alone in the Raw264.7 cells. Second, MKP-1 expression was increased by *S. aureus* stimulus and it was further enhanced in the presence of rolipram and other cAMP-elevating agents (forskolin and 8-Bromo-cAMP) in Raw264.7 cells. Additionally, rolipram could inhibit *S. aureus* induced phosphorylations of p38 and IκBα. Furthermore, our previous data demonstrated that MKP-1 could be an endogenous inhibitor of both p38 and IκBα molecules by *S. aureus* stimulus in Raw264.7 cells. Taken together, these observations suggested that the anti-inflammatory effect of rolipram on *S. aureus* induced inflammation could be mediated by MKP-1 *via* a cAMP-PKA dependent pathway.

Another interesting finding in our study was PKA-cα could directly associate with IκBα by *S. aureus* stimulus. In the current study, *S. aureus* treatment induced a high level expression of PKA-cα and activation of IκBα. Additionally, a block of PKA activity by KT 5720 remarkably enhanced the activation of IκBα induced by *S. aureus*. Furthermore, pretreated with rolipram and cAMP-elevating agents were all capable of inhibiting *S. aureus* induced IκBα activation in Raw264.7 cells. These data indicated that there was the crosstalk between IκBα and cAMP-PKA pathways during *S. aureus* stimulation. A direct link between IκBα and PKA pathway was established through the finding that PKA-cα could directly associate with IκBα in response to *S. aureus*. In our studies, PKA-cα was immunoprecipitated with IκBα in the resting Raw264.7 cells. When stimulated with *S. aureus*, this interaction was significantly reduced, disrupted the IκBα/PKA-cα complex. These results were consistent with the former studies that PKA-c resides constitutively tethered to IκB/NFκB complexes in the cytoplasm until IκBα was phosphorylated and degraded^30,31,34^. It should be emphasized that IκBα/PKA-cα complex was re-constituted after *S. aureus* stimulated for the later time. The three-dimensional structure of PKA revealed that this protein has a bilobate structure. The smaller N-terminal lobe is primarily used for binding to ATP, while substrate binding is carried out with sequences from the larger C-terminal lobe^35^. It has been testified that N-terminal ATP-binding domain of PKA binds to IκB, while the C-terminal region establishes interaction with the substrate p65 protein. Dual sites of interaction might thus allow PKA bind stably to the IκB/NFκB complex^30^. In our study, the results of pull-down experiments indicated that IκBα may re-constituted with N-terminal ATP-binding domain of PKA-cα and decreased of the PKA enzymatic activity after stimulated with *S. aureus*. These results presented a novel mechanism of PKA and IκB pathway in an auto-regulatory feedback manner. However, there still has a question: how about the C-terminal substrate binding domain? We have tried to use the antibody against PKA-cα to pull-down the P65. Unfortunately, we could not detect apparent band against the P65 (data not shown). We believe that the interaction between PKA-c and IκB might include other regulatory proteins. Identifying other molecules anchored for PKA-c will be an important area of investigation in the future.

Taken together, although we assumed that PKA was required for the induction of MKP-1 by *S. aureus*, we could not discard the involvement of other signaling molecules that have not analyzed in this report. In addition, some studies have implicated that CREB also can act as a p38 and ERK1/2 regulated transcription factor required for macrophage survival during LPS stimulation^18,36^. In fact, we did not get a completely blocking expression of MKP-1 in Raw264.7 cells when treated with the specific inhibitor against PKA at 30 minutes by *S. aureus*. Besides, blocking the activity of PKA was not significantly attenuation the phosphorylation of CREB (Ser133) at 30 minutes by *S. aureus*. These observations suggested that PKA was required for this process but some other mechanisms may participate in the induction of MKP-1 by *S. aureus*. We are currently analyzing the involvement of other pathways that regulate MKP-1 expression.

**Figure 8:**
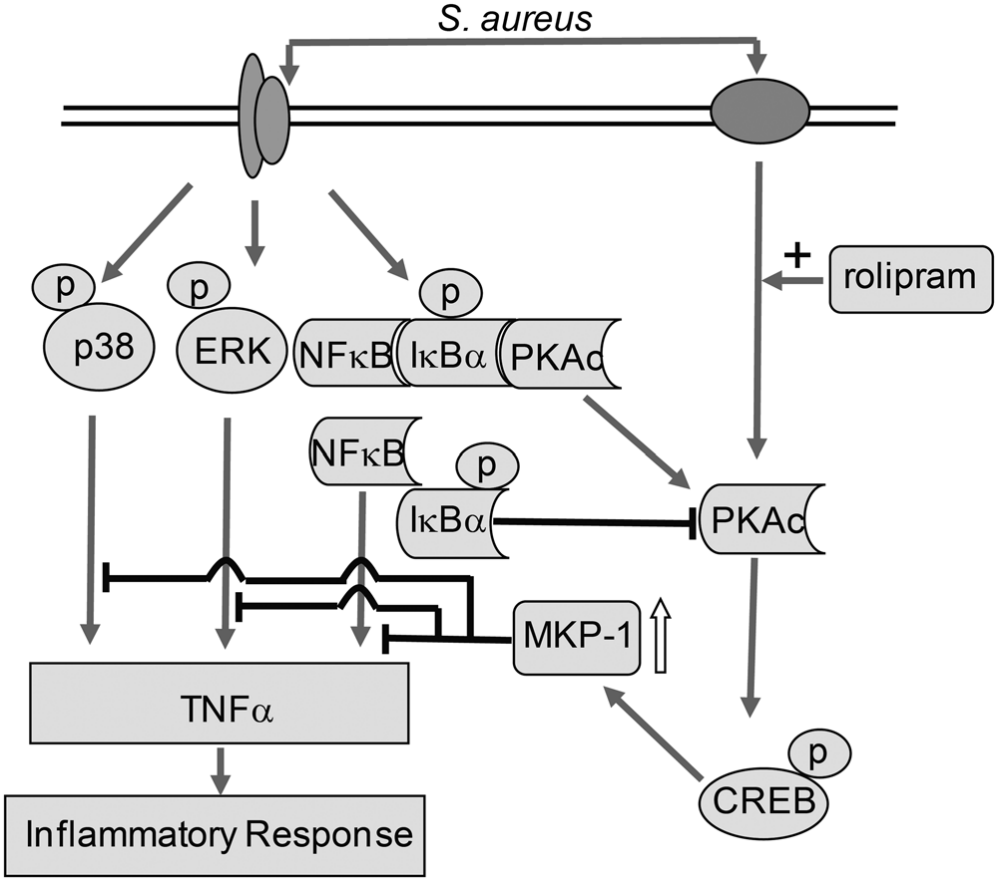
Schematic representation of MKP-1 mediated negative regulation of MAPK and NFκB activations induced by *S. aureus* in Raw264.7 cells.

In summary, our present study demonstrated that MKP-1 was an important endogenous inhibitor of ERK1/2, p38 and IκBα moleculess in response to the Gram-positive bacterium, *S. aureus*. In addition, MKP-1 expression was enhanced by the anti-inflammatory drug, rolipram *via* cAMP-PKA pathway with *S. aureus* stimulation. Furthermore, IκBα could be regulated and directly associate with PKA-cα and this interaction may involved in the anti-inflammatory effect of rolipram by *S. aureus* stimulus (Fig. 8). These findings were of particular therapeutic importance because negative regulators has long been thought as therapeutic strategy during pathogen infection. Therefore, investigation the molecular mechanisms by which MKP-1 was up-regulated may not only bring novel insight into the immune homeostasis but also lead to identification of novel anti-inflammatory drug for controlling inflammation in response to *S. aureus*.

## Materials and Methods

### Reagents

The following agents U 0126, SB 203580, Bay 11-7082, KT 5720 (Calbiochem Corp, USA) and rolipram, foskolin (Sigma-Aldrich, USA) were dissolved in DMSO. Final concentration of DMSO was less than 0.1%. 8-Bromo-cAMP (8-Bromoadenosine 3′,5′-cyclic monophosphate sodium salt) was purchased from Sigma-Aldrich. MKP-1, total-IkBα, total-ERK2 antibodies obtained from Santa Cruz Biotechnology. MKP-3, MKP-5, phospho-IkBα (Ser32/36), phospho-p38 (Thr180/Tyr182), total-p38, phospho-ERK1/2 (Thr202/Tyr204), phospho-CREB (Ser133) and PKA-cα were purchased from Cell Signaling Technology. β-actin was purchased from Sigma-Aldrich. Dulbecco’s modified Eagle’s medium (DMEM), penicillin and streptomycin were from Invitrogen Life Technologies. All other reagents were purchased from Sigma-Aldrich, unless indicated otherwise.

### *S. aureus* preparation and infection procedure

*S. aureus* was grown overnight at 37 °C in a shaking incubator (200 rpm) in tryptic soy broth (TSB) (Sigma). Bacteria was harvested by centrifugation at 800 × g for 10 minutes, washed twice with sterile PBS (Invitrogen, USA) and resuspended in PBS. The optical density (OD) was determined for *S. aureus* number estimation (A_600_: 0.8 OD unit ≈ 2 × 10^8^ cfu/ml). *S. aureus* was added into DMEM without penicillin and streptomycin to get a final concentration 2 × 10^7^ cfu/ml. The Raw264.7 cells monolayers (80~90% confluency) were washed and changed to no penicillin/streptomycin medium containing 2 × 10^7^ cfu/ml *S. aureus*. The cells were harvested at different time points after infection.

### Cell culture and drugs

Raw264.7 cells were cultured in DMEM supplemented with 10% fetal calf serum, 100 U/ml of penicillin G and 100 μg/ml streptomycin in a humidified 37 °C incubator. Prior to experimentation, cells were seeded on six well plate (2 × 10^5^ per well) and incubated overnight, then changed to fresh no penicillin/streptomycin medium for prescribed periods of time in the presence or absence of pharmacological inhibitors or reagents.

### siRNA (small interfering RNA)-mediated gene silencing of *Mkp-1*

Raw264.7 cells stably harboring the previously described *Mkp-1* siRNA were provided by Dr. Yusen Liu^37^. Briefly, Raw264.7 cells were transfected with *Mkp-1* siRNA expression plasmid, using FuGENE® 6 Transfection Reagent (Roche Diagnostics, USA) according to the manufacturer’s recommendations. After transfection, cells were selected in medium containing 500 μg/ml of G418 (Roche Diagnostics, USA) for two weeks, and isolated the individual G418-resistant clones. These clones were screened for MKP-1 expression with western-blot analyses by using antibody against MKP-1. Stable Raw264.7 clones expression *Mkp-1* siRNA were maintained in medium containing 100 μg/ml of G418.

### RNA isolation, cDNA synthesis and SYBR Green Real Time PCR

Total RNA was isolated using TRIzol® reagent (Invitrogen, USA) according to the manufacturer’s protocols, and reverse transcription reaction was prepared using 1 μg of RNA to abtain cDNA (Qiagen, USA). The resultant cDNA was diluted 1:3 in RNAse-free water. Realtime quantitative PCR (q-PCR) was performed using ABI 7700 Sequence Detection System (Applied Biosystems, USA) as described^38–40^. The sequence of primers used were as follows: mouse *Mkp-1*: sense, 5′-ACC ATC TGC CTT GCT TAC CTT-3′; antisence: 5′-AGC ACC TGG GAC TCA AAC TG-3′; mouse *TNFα*: sense, 5′-GAC CCT CAC ACT CAG ATC ATC TTC-3′; antisence: 5′-CAC GTA GTC GGG GCA GCC TTG-3′; mouse *β-actin*: sense, 5′-GAA GAG CTA TGA GCT GCC TGA-3′; antisence: 5′-CAG CAC TGT GTT GGC ATA GAG-3′.

### Protein preparation, Immunoprecipitation and Western blotting

Whole cell lysates were prepared using cell lysis buffer containing 10 mM HEPES (pH 7.4), 50 mM β-glycerophosphote, 1% Triton X-100, 1% NP-40, 10% glycerol, 2 mM EDTA, 2 mM EGTA, 1 mM DTT, 10 mM NaF, 1 mM Na_3_VO_4_ and complete protease inhibitor cocktail (Roche Diagnostics). The lysates were centrifuged and the supernatants were boiled in SDS loading buffer. For immunoprecipitation, cell lysates were incubated with appropriate amount of antibody overnight and then precipitated following absorption onto protein A-agarose. Precipitates were washed three times, separated by SDS-PAGE, then transferred onto nitrocellulose membrane which was then incubated in TBST buffer (150 mM NaCl, 20 mM Tris-HCl, and 0.02% Tween 20, pH 7.6) containing 5% non-fat milk. Western blot analysis was conducted by using ECL reagent (Pierce, USA). The membranes probed with phospho-IkBα, phospho-p38 and phospho-ERK1/2 were stripped and re-probed with antibodies against total IkBα or total p38 or total ERK2 to confirm equal sample loading.

### Statistical analysis

One-way ANONA was used to assess significant differences among treatment groups. For each significant effect of treatment, SPSS statistical software program was used for comparisons of multiple group means. A value of *p* < 0.05 was considered significant.

## Electronic supplementary material

Supplementary data
