# Supplementary material for: MKP-1 negative regulates Staphylococcus aureus induced inflammatory responses in Raw264.7 cells: roles of PKA-MKP-1 pathway and enhanced by rolipram
Source: Sci Rep. 2017 Sep 28;7:12366. doi: 10.1038/s41598-017-10187-3 (PMC5620070; doi:10.1038/s41598-017-10187-3)
Supplement: Supplementary file 1 — Supplementary data [file 41598_2017_10187_MOESM1_ESM.pdf]

MKP-1 negative regulates *Staphylococcus aureus* induced inflammatory responses in Raw264.7 cells: roles of PKA-MKP-1 pathway and enhanced by rolipram

Yiqing Pan, Chen Xu & Zhixing K. Pan

Supplementary data

Materials and methods

Measurement of intracellular cAMP. The concentration of cAMP was measured using a bioluminescence assay kit. Briefly, Raw264.7 cells monolayers (~90% confluency) were treated with rolipram (10 μM), forskolin (10 μM) and 8-Bromo-cAMP (250 μM) for 5, 15 and 60 minutes respectively. The cells were harvested using sample diluents supplied in the kit. Aliquots (50 μl) of the supernatants were assayed for cAMP using bioluminescence assay kit (cAMP-Glo™ Assay, Promega Corporation, USA) according to the manufacturer's instructions. cAMP values were read using a microplate-reading luminometer. Luminescence can be correlated to the cAMP concentrations by using a cAMP standard curve.

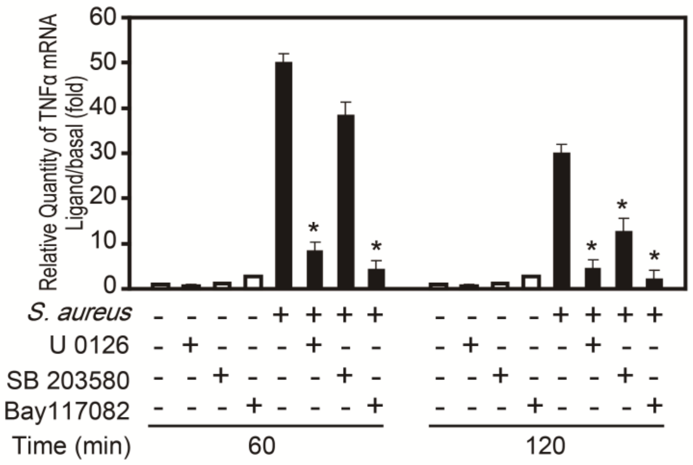

Figure S1 Effects of the U0126, SB203580 and Bay 117082 on TNFα production induced by *S. aureus* in Raw264.7 cells. Raw264.7 cells were incubated with U 0126 (10 μM), SB203580 (10 μM) and Bay 11-7082 (10 μM) as indicated for 30 minutes prior to stimulation with *S. aureus* (10 M.O.I.) or control. Cells were harvested for 60 to 120 minutes, real-time PCR of *TNFα* expression was carry out. ∗ p<0.05.

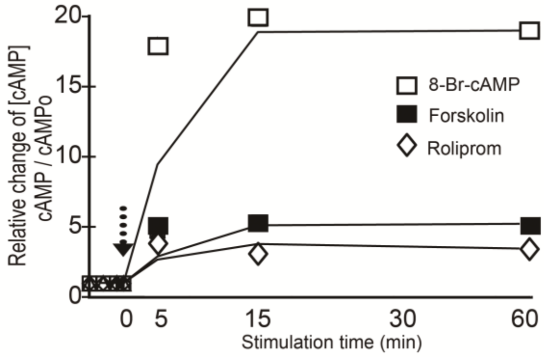

Figure S2 Effects of rolipram, foskolin and 8-Bromo-cAMP on the intracellular cAMP concentrations in Raw264.7 cells. Raw264.7 cells were treated with rolipram (10 μM), foskolin (10 μM) and 8-Bromo-cAMP (250 μM) for 5, 15, 30 and 60 minutes respectively. The cells that were harvested and aliquots (50 μl) of the supernatants were assayed for cAMP using cAMP-GloTM Assay according to the manufacturer's instructions. cAMP values were read and correlated to the cAMP concentrations by using a cAMP standard curve.
